# Psychotropic Medication Use Is Associated With Greater 1-Year Incidence of Dementia After COVID-19 Hospitalization

**DOI:** 10.3389/fmed.2022.841326

**Published:** 2022-03-18

**Authors:** Yun Freudenberg-Hua, Alexander Makhnevich, Wentian Li, Yan Liu, Michael Qiu, Allison Marziliano, Maria Carney, Blaine Greenwald, John M. Kane, Michael Diefenbach, Edith Burns, Jeremy Koppel, Liron Sinvani

**Affiliations:** ^1^Department of Psychiatry, Zucker Hillside Hospital, Northwell Health, Glen Oaks, NY, United States; ^2^The Feinstein Institutes for Medical Research, Northwell Health, Manhasset, NY, United States; ^3^Donald and Barbara Zucker School of Medicine at Hofstra/Northwell, Hempstead, NY, United States; ^4^Department of Medicine, Donald and Barbara Zucker School of Medicine at Hofstra Northwell, Hempstead, NY, United States

**Keywords:** COVID-19, dementia, cognitive impairment, post-COVID, psychotropic medication, geriatric

## Abstract

**Background:**

COVID-19 has been associated with an increased risk of incident dementia (post-COVID dementia). Establishing additional risk markers may help identify at-risk individuals and guide clinical decision-making.

**Methods:**

We investigated pre-COVID psychotropic medication use (exposure) and 1-year incidence of dementia (outcome) in 1,755 patients (≥65 years) hospitalized with COVID-19. Logistic regression models were used to examine the association, adjusting for demographic and clinical variables. For further confirmation, we applied the Least Absolute Shrinkage and Selection Operator (LASSO) regression and a machine learning (Random Forest) algorithm.

**Results:**

One-year incidence rate of post-COVID dementia was 12.7% (*N* = 223). Pre-COVID psychotropic medications (OR = 2.7, 95% CI: 1.8–4.0, *P* < 0.001) and delirium (OR = 3.0, 95% CI: 1.9–4.6, *P* < 0.001) were significantly associated with greater 1-year incidence of post-COVID dementia. The association between psychotropic medications and incident dementia remained robust when the analysis was restricted to the 423 patients with at least one documented neurological or psychiatric diagnosis at the time of COVID-19 admission (OR = 3.09, 95% CI: 1.5–6.6, *P* = 0.002). Across different drug classes, antipsychotics (OR = 2.8, 95% CI: 1.7–4.4, *P* < 0.001) and mood stabilizers/anticonvulsants (OR = 2.4, 95% CI: 1.39–4.02, *P* = 0.001) displayed the greatest association with post-COVID dementia. The association of psychotropic medication with dementia was further confirmed with Random Forest and LASSO analysis.

**Conclusion:**

Confirming prior studies we observed a high dementia incidence in older patients after COVID-19 hospitalization. Pre-COVID psychotropic medications were associated with higher risk of incident dementia. Psychotropic medications may be risk markers that signify neuropsychiatric symptoms during prodromal dementia, and not mutually exclusive, contribute to post-COVID dementia.

## Introduction

Emerging literature suggests that neurological and psychiatric disorders are common complications of Coronavirus Disease 2019 (COVID-19), caused by the Severe Acute Respiratory Syndrome Coronavirus 2 (SARS-CoV-2) infection (1, 2). COVID-19 has also been associated with an increased risk of incident dementia (post-COVID dementia) (3–5). Taquet et al. found that the 6-month post-COVID dementia incidence was 2-fold higher than the incidence following influenza (4). As typical in dementia syndromes, this risk was predominantly seen in older adults and appeared to be correlated with disease severity, as those requiring hospitalization for COVID-19 had higher rates of subsequent dementia (4, 5).

Identification of risk markers associated with post-COVID dementia is important to guide clinical decision-making as well as future research on preventative measures in at-risk individuals. Recent large studies using electronic health record (EHR) data have investigated the interplay of psychiatric illness and COVID-19. Their findings indicate that not only does COVID-19 have profound mental health sequelae for those previously healthy, but pre-existing psychiatric illness was associated with worse short-term outcomes including mortality (6, 7). Yet, these studies did not evaluate the use of pre-COVID psychotropic medications.

In non-COVID studies there is conflicting evidence regarding the association between psychotropic medications and dementia risk (8–11). Interestingly, several COVID-related studies have even reported potentially beneficial effects of psychotropic medications (e.g., antidepressants) in preventing SARS-CoV-2 infection, severe illness, and mortality (12–14). Given that psychotropic medications are broadly prescribed among older adults (15–17) and their use is potentially modifiable, the role of psychotropic medications in incident post-COVID dementia requires systematic investigation.

In this article, we leveraged EHR data of 1,755 older adults (≥65 years) hospitalized for COVID-19 to: (1) determine the 1-year incidence rate of post-COVID dementia; (2) assess the association between pre-COVID psychotropic medication use and post-COVID incident dementia; and (3) explore the association between different classes and types of psychotropic medications and post-COVID incident dementia.

## Methods

### Study Design and Population

This retrospective cohort study was performed within Northwell Health, a large integrated academic health system in the greater New York metropolitan area, and conducted following the statement of Standard Reporting of Observational Studies in Epidemiology (STROBE) (18) (Supplementary Table 1). The study protocol was approved by the local Institutional Review Board.

**Figure 1:**
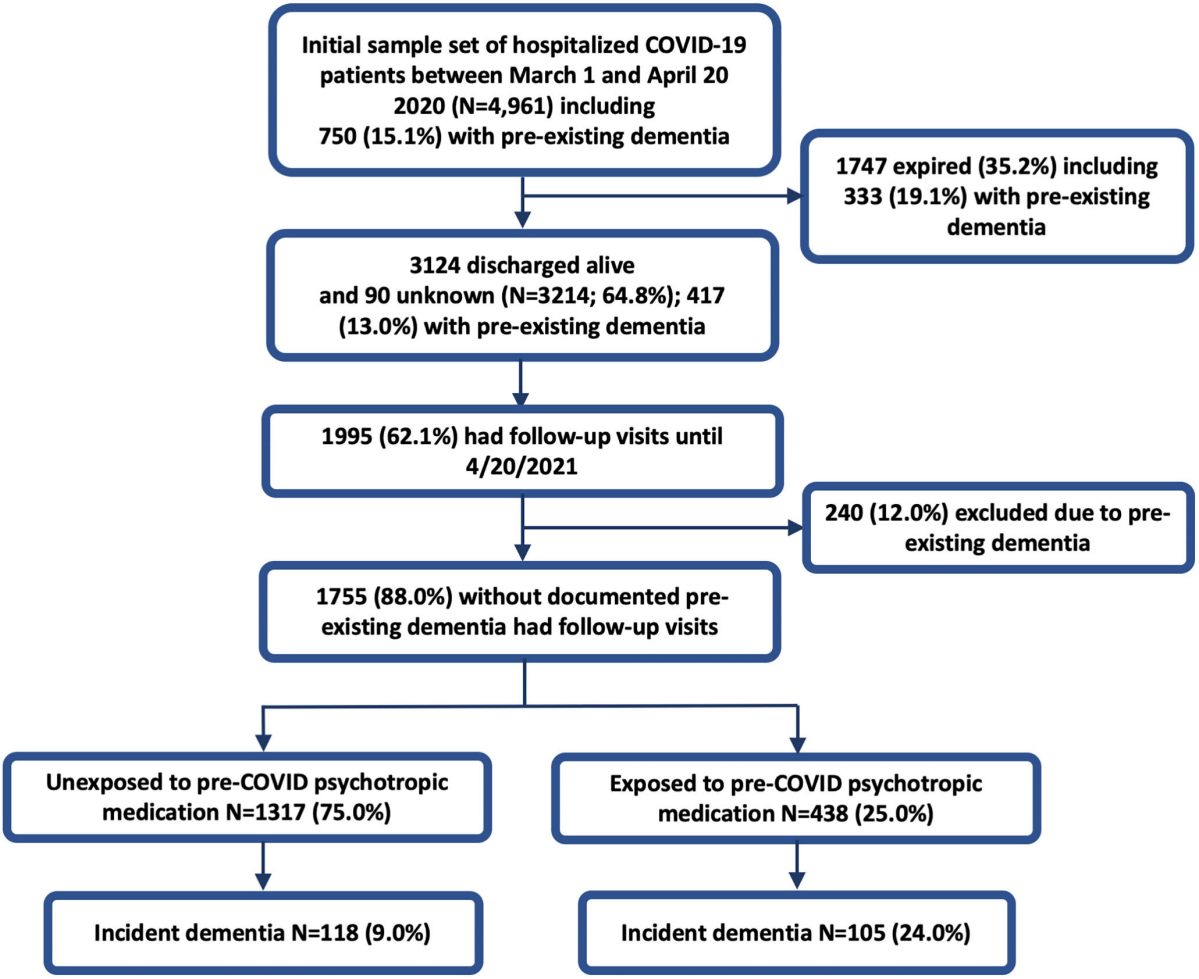
Study cohort.

Demographic and clinical data was extracted from the EHR by the data management team. We included adults aged 65 years or older, who were hospitalized in one of 11 health system hospitals between March 1st, 2020 and April 20th, 2020, with a positive SARS-CoV-2 test result (19). Index COVID-19 hospitalization was defined as the first admission with a SARS-CoV-2 infection confirmed by a positive reverse-transcriptase–polymerase-chain-reaction (RTPCR) test in nasopharyngeal specimens. Inclusion for this study required that patients were discharged alive after their index admission and had at least one follow-up visit within the health system (outpatient, inpatient, or emergency department) by April 20th, 2021. Patients were excluded if they had a previous diagnosis of dementia or cognitive impairment [International Classification of Diseases, Tenth Revision, Clinical Modification (ICD-10) codes starting with F01, F02, F03, F04, F09, G30, G31, G32, and R41] prior to the index admission or if they were prescribed a medication used for dementia (Supplementary Table 2; Figure 1).

### Outcome Variable

The primary outcome was 1-year incident dementia, defined as either of the following: (1) any new diagnosis of dementia or cognitive impairment (ICD-10 codes starting with F01, F02, F03, F04, F05, F09, G30, G31, G32, and R41) that was recorded in the EHR during any follow-up visit until April 20th, 2021; and/or (2) a new prescription for medications used for dementia (Supplementary Table 2). One-year incidence of post-COVID dementia was compared between patients with documented use of at least one psychotropic medication prior to the index COVID-19 hospitalization (pre-COVID) vs. patients without pre-COVID psychotropic medication use (defined below).

### Psychotropic Medication Use, Demographic and Clinical Variables

Pre-COVID psychotropic medication use was defined as any documented prescription before the index COVID-19 hospitalization (Supplementary Table 2). The list included antipsychotics, antidepressants, anticonvulsants and mood stabilizers (including lithium), benzodiazepines, and antiparkinson medications. We extracted pre-COVID psychotropic “home medications” as recorded in the “admission medication reconciliation” document, as well as those prescribed within the first 24 h of the index admission. During the initial pandemic peak, home medications were often entered directly into the hospital orders rather than being reconciled separately in the appropriate location. The “home medications” filled at registered pharmacies in New York were automatically populated into the EHR leading to comprehensive documentation. Based on the guiding pharmacological principles of treating delirium in acutely medically ill (20), new psychotropic medications were not initiated as standing medication to treat delirium in the first 24 h. Psychotropic medications ordered “as needed” (i.e., pro re nata) for behavioral symptoms during index admission were excluded. Neurological and psychiatric diagnoses (ICD-10 F codes and G codes, I6x, and R56) were extracted from “past medical history” of index admission.

Demographic variables included age, sex, chart documented race (Asian, Black, White, “other or unknown”), and ethnicity (Hispanic or Other). Patient characteristics included dichotomized smoking history (“current or formal smoker” or “never smoker”); body mass index (BMI), which was calculated from weight and height, using the formula BMI = weight (kg)/height (m)^2^; and comorbidity index, which was derived using the Charlson Comorbidity Index without the age component of the score (21, 22). BMI < 14 (below one percentile of our data; *N* = 20) was regarded as missing, due to likely data input errors. Clinical variables representing severity of the acute COVID-19 illness included the Modified Early Warning Score (MEWS) (23) at the time of admission and highest level of oxygen requirement during index hospitalization. The MEWS is an objective score designed to assess severity of illness (23). The highest level of oxygen support during the index COVID-19 admission were collapsed into three categories (in increasing order of severity): “0” for room air or nasal cannula; “1” for Venturi Mask, non-rebreather, high flow, and non-invasive positive-pressure ventilation; “2” for invasive mechanical ventilation. Delirium during the index COVID-19 hospitalization was defined as ICD10 code F05.

### Statistical Analysis

We applied several types of analyses to investigate the association between predictor variables and post-COVID incident dementia. First, unadjusted analyses included Fisher's exact or Chi-square tests for categorical factors as well as *t*-test or its non-parametric version (Wilcoxon test), if needed, for continuous variables. Second, multivariable (multiple) logistic regression with corresponding variable selection was utilized. Multivariable logistic regression was also performed in the subgroup including only patients with at least one documented history of neurological or psychiatric diagnosis. For Cox proportional hazard ratio model, we calculated time-to-event (days) based on time point of index COVID-19 admission, time point of first dementia diagnosis and time point of last follow-up visit without a dementia diagnosis. Two additional methods were applied to confirm the robustness of multiple regression results. First, we applied a machine learning algorithm—random forest (RF) (24) to identify the importance of the predictor variables for outcome prediction (25). RF has been frequently used in EHR data due to its robustness to noise, such as collinearity, in high dimensional data (26, 27). Next, we applied Least Absolute Shrinkage and Selection Operator (LASSO) regression to minimize overfitting of variables (28) (Supplementary Material). Using these performance-based methods to evaluate input variables can be considered as an alternative to the *p*-values in a regression model (29). Lastly, in secondary exploratory analyses, we evaluated associations between individual psychotropic medications and post-COVID dementia using univariable Fisher's exact test.

All statistical analyses were carried out on the R platform (version 4.0.3) (30) (https://www.r-project.org). The R statistical packages used for this study include glmnet package for LASSO, randomForest package for RF and imputation for missing values, and tableone (31) for presentation. A power analysis for testing the association of an individual medication with the outcome was performed using two-sample test for proportions with unequal sample sizes and implemented in pwr package (32).

## Results

### Incidence of Post-COVID Dementia and Unadjusted Analysis

Of the 4,961 older adults hospitalized with COVID-19 between March 1st, 2020 to April 20th, 2020 that were previously reported (19), 1,755 patients (mean age 75.3 years) without pre-COVID dementia had at least one follow-up visit in Northwell within 1-year following the index COVID-19 admission (Figure 1; Table 1). A total of 223 (12.7%) patients developed incident dementia within 1-year follow-up. Of the 438 (25.0%) patients exposed to at least one pre-COVID psychotropic medication, 105 (24.0%) developed dementia, compared to 118 (9.0%) of the 1,317 patients without the exposure (OR = 3.20, 95% CI: 2.37-−4.32, Fisher's exact *P* = 1.19 × 10^−14^) who developed dementia. Among the psychotropic medication users, 121 were on antipsychotics, 244 on antidepressants, 110 on benzodiazepines, 95 on mood stabilizers or anticonvulsants, and 46 on antiparkinson medications. Univariable analysis (Table 1) demonstrated that age, BMI, Comorbidity Index, delirium during index COVID-19 admission, prior use of antipsychotics, antidepressants, benzodiazepines and mood stabilizers/anticonvulsants, were significantly associated with a higher risk of incident post-COVID dementia.

**Table 1:** Patient demographics and characteristics stratified by post-COVID incident dementia (unadjusted).

**Variable**	**Level**	**No incident dementia**	**Incident dementia**	***P*** **-value**	**Missing (%)**
*N*		1,532	223		
Sex (%)	F	669 (43.7)	103 (46.2)	0.53	0
	M	863 (56.3)	120 (53.8)		
Age [mean (SD)]		74.85 (7.46)	78.40 (8.02)	<0.001	0
Race (%)	Asian	103 (6.7)	13 (5.8)	0.05	0
	Black	389 (25.4)	43 (19.3)		
	Other or unknown	362 (23.6)	47 (21.1)		
	White	678 (44.3)	120 (53.8)		
Ethnicity (%)	Hispanic	237 (16.2)	27 (12.6)	0.2	4.6
	Non-hispanic	1,222 (83.8)	188 (87.4)		
Smoking (%)	No	1,408 (93.9)	196 (92.9)	0.69	2.5
	Yes	92 (6.1)	15 (7.1)		
BMI [mean (SD)]		27.61 (5.94)	25.47 (6.05)	<0.001	4.1
Comorbidity index [mean (SD)]		3.25 (2.85)	4.12 (3.00)	<0.001	0.1
MEWS [mean (SD)]		3.48 (1.16)	3.63 (1.30)	0.13	18.9
Highest oxygen delivery (%)	0	1,068 (69.7)	162 (72.6)	0.01	0
	1	319 (20.8)	34 (15.2)		
	2	145 (9.5)	27 (12.1)		
Delirium (%)	No	1,351 (88.2)	151 (67.7)	<0.001	0
	Yes	181 (11.8)	72 (32.3)		
Antipsychotic (%)	No	1,449 (94.6)	185 (83.0)	<0.001	0
	Yes	83 (5.4)	38 (17.0)		
Antidepressant (%)	No	1,345 (87.8)	166 (74.4)	<0.001	0
	Yes	187 (12.2)	57 (25.6)		
Benzodiazepine (%)	No	1,444 (94.3)	201 (90.1)	0.03	0
	Yes	88 (5.7)	22 (9.9)		
Mood stabilizer/ anticonvulsant (%)	No	1,468 (95.8)	192 (86.1)	<0.001	0
	Yes	64 (4.2)	31 (13.9)		
Antiparkinson (%)	No	1,496 (97.7)	213 (95.5)	0.1	0
	Yes	36 (2.3)	10 (4.5)		
Any psychotropic (%)	No	1,199 (78.3)	118 (52.9)	<0.001	0
	Yes	333 (21.7)	105 (47.1)		

*Ethnicity, Hispanic or not-Hispanic; Smoking, “No” is defined as never smoker and “Yes” as current or past smoker; BMI, body mass index; Comorbidity Index, Charlson Comorbidity Index without the age component; MEWS, Modified Early Warning Score. Highest Oxygen Delivery: the highest level of oxygen support during index hospitalization. “0”: room air or nasal cannula; “1”: Venturi Mask, non-rebreather, high flow, non-invasive positive-pressure ventilation; “2”: invasive mechanical ventilation. Any Psychotropic: medications including all subcategories of psychotropic medications: antipsychotics, antidepressants, benzodiazepines, mood stabilizers/anticonvulsants (including lithium), and Parkinson's disease medications (Antiparkinson)*.

### Multiple Regression Analysis

We applied three multiple regression models. **Model 1** examined the relationship between “any psychotropic medication” use and post-COVID dementia, adjusting for age, gender, race, ethnicity, BMI, smoking history, Comorbidity Index, MEWS, highest level of oxygen requirement, and delirium. Pre-COVID use of any psychotropic medication was significantly associated with post-COVID dementia (OR = 2.68, 95% CI: 1.79–4.01, *P* < 0.001) (Table 2, Model 1). Model 2 examined the association of each class of psychotropic medications (i.e., antipsychotics, antidepressants, mood stabilizers/anticonvulsants, benzodiazepines, and antiparkinson medications listed in Supplementary Table 2), adjusting for all variables in Model 1, except for “any psychotropic medication.” After adjusting, antipsychotics and mood stabilizers/anticonvulsants remained significant (Table 2, Model 2). Model 3 included all variables that were significant at *P* ≤ 0.05 in Model 2. In Model 3 (Table 2), antipsychotic medications (OR = 2.75, 95% CI: 1.69–4.38, *P* < 0.001) and mood stabilizers/anticonvulsants (OR = 2.39, 95% CI: 1.39–4.02, *P* = 0.001) were associated with a more than 2-fold increased risk of incident post-COVID dementia. Additionally, delirium during the index COVID-19 admission was associated with a 2-fold increased risk of incident post-COVID dementia (OR = 2.36, 95% CI: 1.65–3.35, *P* < 0.001). Model 3 also demonstrated that each additional year of age was associated with a 5% increase of post-COVID dementia (OR = 1.05, 95% CI: 1.03-−1.07, *P* < 0.001) and each unit increase of the comorbidity index was associated with a 6% increased risk of post-COVID dementia (OR = 1.07, 95% CI: 1.02–1.12, *P* = 0.009). On the other hand, each unit increase of BMI was associated with a 4% decreased risk of post-COVID dementia (OR = 0.96, 95% CI: 0.93-−0.99, *P* = 0.003) (Table 2). Furthermore, we analyzed hazard ratios (HR) for incident dementia using days between the index COVID-19 hospitalization and dementia diagnosis for the variables in Model 1 and Model 2 (Supplementary Table 3). The results of HRs are comparable with the ORs.

**Table 2:** Multivariable logistic regression for variables associated with 1-year incident dementia in older adults hospitalized with COVID-19 (*n* = 1,755).

	**Model 1**			**Model 2**			**Model 3**		
**Predictors**	**OR**	**95% CI**	***p***	**OR**	**95% CI**	***p***	**OR**	**95% CI**	***p***
Age	1.03	1.00 – 1.06	**0.04**	1.04	1.01 – 1.07	**0.01**	1.05	1.03 – 1.07	**<0.001**
Sex (ref = “female”)	0.84	0.57 – 1.24	0.37	0.83	0.56 – 1.22	0.34	___	___	___
Race, Asian (ref = “white”)	1.09	0.47 – 2.29	0.83	1.07	0.46 – 2.24	0.87	___	___	___
Race, Black (ref = “white”)	0.68	0.40 – 1.12	0.14	0.66	0.39 – 1.10	0.12	___	___	___
Race, other/unknown (ref = “white”)	1.17	0.61 – 2.18	0.63	1.19	0.62 – 2.24	0.59	___	___	___
Ethnicity (ref = “hispanic”)	1.13	0.57 – 2.31	0.74	1.1	0.55 – 2.25	0.80	___	___	___
Smoking	0.86	0.39 – 1.73	0.69	0.88	0.40 – 1.78	0.74	___	___	___
BMI	0.96	0.93 – 1.00	**0.04**	0.96	0.93 – 1.00	**0.04**	0.96	0.93 – 0.99	**0.003**
Comorbidity index	1.07	1.00 – 1.14	0.06	1.07	1.00 – 1.14	**0.04**	1.07	1.02 – 1.12	**0.009**
Delirium	3.01	1.94 – 4.63	**<0.001**	2.78	1.77 – 4.32	**<0.001**	2.36	1.65 – 3.35	**<0.001**
MEWS	0.99	0.82 – 1.19	0.92	1	0.82 – 1.19	0.97	___	___	___
Highest oxygen delivery	1.18	0.88 – 1.57	0.27	1.19	0.88 – 1.58	0.25	___	___	___
Any psychotropic	2.68	1.79 – 4.01	**<0.001**	___	___	___	___	___	___
Antipsychotic	___	___	___	2.27	1.20 – 4.19	**0.01**	2.75	1.69 – 4.38	**<0.001**
Mood stabilizer/ anticonvulsant	___	___	___	2.48	1.25 – 4.72	**0.007**	2.39	1.39 – 4.02	**0.001**
Antidepressant	___	___	___	1.59	0.97 – 2.53	0.06	___	___	___
Benzodiazepine	___	___	___	1.36	0.66 – 2.64	0.39	___	___	___
Antiparkinson	___	___	___	1.36	0.47 – 3.38	0.54	___	___	___

*OR, odds ratio; For binary variables Smoking, Delirium, Any Psychotropic, Antipsychotic, Mood Stabilizer/Anticonvulsant, Antidepressant, Benzodiazepine, and Antiparkinson, the reference values were “No” or “not exposed.” Ethnicity, Hispanic or not-Hispanic; Smoking, never smoker or current/past smoker; BMI, body mass index; Comorbidity Index, Charlson Comorbity Index; MEWS, Modified Early Warning Score. Highest Oxygen Delivery: Highest level of oxygen support during index hospitalization; Any Psychotropic: medications including all subcategories of psychotropic medications: antipsychotics, antidepressants, benzodiazepines, mood stabilizers/anticonvulsants (including lithium), and Parkinson's disease medications (Antiparkinson). Significant P values are in bold*.

We further performed a sensitivity analysis in the subset of 423 patients who had at least one neurological and psychiatric diagnosis that were documented during the index COVID-19 hospitalization, adjusting for all variables in **Model 1**. Pre-COVID psychotropic medication use remained significantly associated with post-COVID dementia (OR = 3.09, 95% CI: 1.52–6.57, *P* = 0.002; Supplementary Table 4).

### Secondary Unadjusted Analyses

In exploratory secondary unadjusted analyses, we examined the unadjusted association between individual psychotropic medications and post-COVID dementia (see sample size power analysis in Supplementary Figure 1). Among the top 20 most frequently prescribed medications in our cohort, valproic acid (OR = 11.57, 95% CI: 3.59-−38.18, *P* < 0.001), haloperidol (OR = 8.44, 95% CI: 3.19–21.83, *P* < 0.001), mirtazapine (OR = 6.02, 95% CI: 3.12-−11.34, *P* < 0.001), levetiracetam (OR = 5.91, 95% CI: 3.17-−10.80, *P* < 0.001), clonazepam (OR = 3.97, 95% CI: 1.58-−9.16, *P* = 0.002), quetiapine (OR = 3.9, 95% CI: 1.64-−8.61, *P* = 0.001), and escitalopram (OR = 3.49, 95% CI: 1.54-−7.33, *P* = 0.002) were significantly associated with increased risk for post-COVID dementia (at Bonferroni corrected *P*-value threshold of <0.0025) (Table 3).

**Table 3:** Association of individual psychotropic medications with post-COVID dementia.

**Medication**	**Counts**	**Odds ratio**	**95% CI**	***P***
Levetiracetam	57	**5.91**	**3.17 – 10.80**	**<0.001**
Mirtazapine	51	**6.02**	**3.12 – 11.34**	**<0.001**
Sertraline	51	2.48	1.08 – 5.19	0.02
Alprazolam	46	0.97	0.25 – 2.74	1
Escitalopram	43	**3.49**	**1.54 – 7.33**	**0.002**
Quetiapine	36	**3.9**	**1.64 – 8.61**	**0.001**
Carbidopa-Levodopa	35	3.51	1.41 – 7.96	0.004
Clonazepam	32	**3.97**	**1.58 – 9.16**	**0.002**
Trazodone	28	2.77	0.90 – 7.22	0.04
Olanzapine	25	3.2	1.03 – 8.55	0.02
Haloperidol	22	**8.44**	**3.19 – 21.83**	**<0.001**
Risperidone	22	2.98	0.85 – 8.63	0.04
Duloxetine	21	2.39	0.58 – 7.49	0.12
Lorazepam	21	3.17	0.89 – 9.266	0.04
Paroxetine	18	2.03	0.37 – 7.33	0.22
Bupropion	16	3.38	0.78 – 11.38	0.05
Citalopram	16	1.45	0.17 – 6.44	0.65
Valproic acid	15	**11.57**	**3.59 – 38.18**	**<0.001**
Aripiprazole	14	2.77	0.49 – 10.68	0.13
Fluoxetine	13	1.85	0.20 – 8.61	0.33

*Counts: number of patients taking the medication. Bonferroni corrected P-value significance threshold is 0.0025 for 20 univariable associations (Fisher's exact test). Significant P values are in bold*.

The lack of association of post-COVID dementia with COVID-19 severity, as measured by MEWS and highest level of oxygen delivery, is counterintuitive. We therefore asked whether MEWS/oxygen delivery (e.g., information on COVID-19 severity) is to a certain extent included in the delirium variable. In unadjusted analysis, MEWS were associated with delirium (OR = 1.19, *P* = 0.004), but this association could be explained by age (OR = 1.09, *P* = 0.24 after adjusting for age). Levels of oxygen support was not associated delirium (OR = 1.14, *P* = 0.20) in unadjusted analysis.

From all patients who did not expire during the index hospitalization (*N* = 3,214), 1995 (62.1%) had available 1-year follow-up data within our health system. We compared the demographic and clinical variables between those with and without follow-ups (potentially seeking care elsewhere; Supplementary Table 5). Among relevant features for post-COVID dementia, significantly higher comorbidity index (3.46 vs. 2.61, *P* < 0.001), higher BMI (27.65 vs. 26.81, *P* = 0.005), as well as higher proportion of exposure to “Any Psychotropic” (30.9 vs. 25.4%, *P* = 0.001) were observed in those with follow-ups. However, antipsychotic medications (8.8 vs. 8.4%, *P* = 0.74) and mood stabilizers/anticonvulsants (6.0 vs. 4.8%, *P* = 0.15), the two classes highly associated with post-COVID dementia, did not differ significantly between the two groups.

### Random Forest (RF) Analyses and LASSO Regression

Next, we applied RF to determine the importance of predictor variables in Model 2, in predicting post-COVID dementia. Using the parameters optimized for unequal sample sizes, the RF model had an out-of-bag (OOB) error rate of 25.41%. The importance of a variable for the prediction accuracy is measured by “Mean Decrease Accuracy,” which represents how much predictive accuracy is lost if a variable is removed from the model. A higher importance score indicates that the variable is more useful for the prediction of incident post-COVID dementia. The model identified the following as the most relevant predictors of incident post-COVID dementia (in declining order of importance): delirium, antipsychotics, mood stabilizers/anticonvulsants, Comorbidity Index, benzodiazepines, and antidepressants (Supplementary Figure 2).

Finally, we applied LASSO regression, which addresses the possibility of overfitting and collinearity of variables. Using the variables in Model 2 the LASSO coefficient profile (Supplementary Figure 3) showed that the predictor variables selected (in declining order) were: delirium, age, antipsychotics, mood stabilizers/anticonvulsants, antidepressants, BMI, and Comorbidity Index. Thus, results from analyses that are less sensitive to collinearity are consistent with our standard regression analysis above and increase confidence in the statistical model.

## Discussion

In this retrospective cohort study of 1,755 older adults hospitalized with COVID-19, the overall 1-year incidence rate of dementia was 12.7%. Pre-COVID psychotropic medication use was associated with higher 1-year incidence of dementia, after controlling for patient demographics, characteristics, and severity of acute COVID-19 illness. To our knowledge, this is the first study to systematically demonstrate an association between pre-COVID psychotropic medications and post-COVID dementia. While recent literature has reported an association between pre-COVID psychiatric illness and post-COVID dementia (6, 7), the use of psychotropic medications until now has been unexplored. Yet, it has been suggested that pre-COVID psychotropic medication use in itself may modulate vulnerability to COVID-19 (33), thereby highlighting the importance of this investigation.

The mechanisms that underlie the observed association between psychotropic medications and post-COVID incident dementia are unknown. It is intuitive that psychotropic medications indicate pre-existing neuropsychiatric conditions in which COVID-19 occurs. It is possible that psychotropic medications may potentiate the neurostructural changes that have been found in the brain of those who have recovered from COVID-19 (34). Our sensitivity analysis in patients with documented neurological and psychiatric diagnoses supports this interpretation. Not mutually exclusive, COVID-19 may have accelerated the underlying brain disorders for which psychotropic medications were prescribed, leading to the greater incidence of post-COVID dementia. In general, severe infections requiring hospitalization had been associated with increased long-term risk of all-cause dementia (35). In pre-COVID literature, the association between psychiatric illness and dementia has been described (11, 36–39). Several hypotheses have been proposed to explain this relationship, including that psychiatric disorders may: share a common pathology with dementia, signify prodromal dementia, or may be an independent risk factor for dementia (36, 40, 41). Further studies are critical to evaluate whether psychotropic medications may serve as a modifiable risk factor for post-COVID dementia.

Within the psychotropic medication classes studied, we found that antipsychotics and anticonvulsants/mood stabilizers were associated with a greater risk of post-COVID dementia. Their relative importance as predictors for post-COVID dementia in this cohort was also highlighted by our machine learning model and LASSO regression analysis. A previous study reported that exposure to antipsychotics in very-late onset schizophrenia-like psychosis was associated with increased dementia risk (38). In contrast, another study showed that antipsychotics were associated with a lower risk of dementia among schizophrenia patients (11). Our results add to these reports regarding associations between antipsychotic use and dementia risk in psychotic disorders. Furthermore, our finding on anticonvulsants and mood stabilizers are consistent with previous literature reporting that regular use of anticonvulsants was associated with a significantly greater risk of incident dementia (42).

In order to gain a deeper understanding of the individual medications that could drive the association signals, we performed explorative analysis on the association of commonly used psychotropic medications (16, 43, 44) with incident post-COVID dementia. We found that valproic acid and haloperidol had the largest effect sizes. Interestingly, valproic acid was associated with an increased risk of dementia in patients with bipolar disorder in a previous non-COVID study (45) and the authors speculated that reduced brain-derived neurotrophic factor expression in the hippocampus lead to less cell proliferation and inhibition of neurite outgrowth (45). To our knowledge, haloperidol use has not been individually linked with increased long-term risk of non-COVID dementia (11, 38). Notably, typical antipsychotics may have an inhibitory effect on SARS-CoV-2 (46), and haloperidol has been previously reported to reduce tau phosphorylation, a hall mark of Alzheimer's dementia, in a mouse model (47). Furthermore, while antidepressants as a class were not associated with post-COVID dementia in our study, the potential effects of two commonly prescribed antidepressants in older adults (16, 48)—mirtazapine and escitalopram—warrant further investigation.

Another new and important finding from our study was the high 1-year incident rate (12.7%) of post-COVID dementia. Al-Aly et al. (5) and Taquet et al. (4) reported a 0.95 and 2.7% 6-month incidence rate of dementia, respectively. However, there are several key differences between these studies and ours: (1) their cohorts included all patients with and without hospitalization, unlike the hospitalized 65+ cohort in our study; (2) the follow-up period was only 6 months, as opposed to 1-year; and (3) they only considered new diagnoses while our study also included new prescriptions of cognitive enhancers such as donepezil as a proxy for new onset of cognitive impairment. In the article by Taquet et al. (4), the 6-month dementia incidence rate for hospitalized patients across all ages was 1.46%, which was 2.2-fold of the incidence (0.67%) for the whole cohort the regardless of hospitalization or age. The authors further reported a 2.66% incidence for patients over the age of 65 regardless of hospitalization, which was 4-fold of the rate for the whole cohort (0.67%). Although the dementia incidence for patients who were both over 65 and hospitalized were not specifically reported, one might extrapolate that it would be around 6% (0.67% × 4 × 2.2) at 6-month, assuming the risk increase is linear. Given the doubled follow-up time in our study, the dementia incidences observed in our study are not inconsistent with their report. The differences in study design likely accounted for the differences of incidence rates but also highlight the importance of focusing on at-risk population.

In our study, the prevalence of pre-existing dementia was 15.1% among all 4,961 COVID-19 hospitalizations, 19.1% among those who expired, and 12.0% among those who had follow-ups. Reynish et al. (49) reported a 13.9% prevalence for dementia or cognitive impairment among people aged 65+ with an emergency medical admission (9.4% known dementia and 4.5% unspecified cognitive impairment). Bellelli et al. (50), employing a formal cognitive assessment, identified a dementia prevalence of 24% in patients aged 65+ in acute hospitals across Italy. The patients in their study were on average older than the patients in our study. The lower dementia prevalence in our follow-up study likely resulted from a combination of factors: (1) survival bias—dementia is associated with the higher COVID-19 mortality (51); (2) age differences; (3) a lack of formal cognitive assessment, which may lead to underestimation of pre-existing cognitive impairment. Moreover, among the patients without pre-existing dementia, the prevalence of antipsychotic medication use was 6.9%. This is higher than those previously reported prevalence of 2–3% among older adults (52, 53). A possible explanation could be that people with severe mental illness have multifold higher odds of hospitalization related to COVID-19 (54).

With regard to other variables found to be associated with post-COVID dementia, we confirmed the previously reported association between delirium and post-COVID dementia (4, 5). In agreement with literature on non-COVID dementia (55), we found that older age was associated with higher incidence of post-COVID dementia. Of interest, COVID-19 severity, as indicated by MEWS and oxygen delivery, was not associated with post-COVID dementia. This might be due to a potential survival bias, as all patients in our study were older and hospitalized (indicating severe COVID-19) and many older adults with severe COVID-19 did not survive their index admissions. Among survivors of COVID-19 hospitalization, MEWS had a weak unadjusted correlation with delirium that could be explained by age and oxygen delivery was not correlated with delirium.

We furthermore investigated the study's validity by comparing the features between patients who had followed up within our healthy system (thus included) and those who did not. While pre-COVID psychotropic exposure was higher among the included subjects, the difference was driven by higher number of antidepressant users, while no significant differences were observed for antipsychotics and anticonvulsants/mood stabilizers—the two drug classes associated with a greater risk of post-COVID dementia.

This study has several strengths. First, we focus on older patients hospitalized with COVID-19, a cohort that appears to be the most vulnerable for the development of post-COVID dementia. Second, our cohort included a diverse patient population with a longer follow-up period then previously reported. Third, our data set included clinically rich variables that underwent vigorous process of data harmonization–ensuring that the data fields represent clinically relevant information. Fourth, we were able to increase the sensitivity of post-COVID dementia detection by not only using documented ICD-10 codes, but also newly started dementia medications. Fifth, our statistical model included not only patient demographics but also a comorbidity score as well as markers of COVID-19 illness severity (MEWS and level of oxygen delivery). Sixth, due to the large sample size, our study was able to systematically investigate different classes of psychotropic medications. Lastly, the robustness of our results was demonstrated by using different statistical methods, including machine learning (Random Forest) and LASSO, which allowed for controlling for overfitting.

This study also has several limitations. First, due to incomplete integration of psychiatric diagnoses in the accessible EHR, a common impediment of EHR studies (56), our study could not comprehensively delineate whether the psychotropic medications or the pre-existing neurological and psychiatric illnesses predicted post-COVID dementia. To infer a causal effect of psychotropic medication use on post-COVID dementia, future studies with comprehensive integration of psychiatric diagnoses are necessary. Second, the method of identifying dementia (EHR documentation and medications) was not amenable to validation given the lack of formal cognitive assessments and could lead to underestimation of pre-existing cognitive impairment. Third, even though the patients were admitted to 11 different hospitals, it still reflects a single health system, which may limit generalizability. Fourth, the associations of individual medications with post-COVID dementia should be interpreted with caution, as the sample sizes did not permit adjustments for covariables. Fifth, no information on lifestyle and economic factors were included, which may influence medication prescription. Lastly, it is also possible that some cases of newly diagnosed dementia could have included long-COVID syndrome, for which cognitive impairment could be part of the clinical presentation (57).

## Conclusion

In this cohort study of older adults hospitalized with COVID-19 at a large health system in New York, exposure to pre-COVID psychotropic medications was associated with greater 1-year incidence of post-COVID dementia. Psychotropic medications may contribute to post-COVID dementia, and not mutually exclusive, serve as a risk marker that signifies neuropsychiatric conditions during prodromal dementia, which was accelerated by COVID-19.

## Data Availability Statement

The original contributions presented in the study are included in the article/Supplementary Materials, further inquiries can be directed to the corresponding author/s.

## Ethics Statement

The studies involving human participants were reviewed and approved by Institutional Review Board of the Feinstein Institutes for Medical Research. Written informed consent for participation was not required for this study in accordance with the national legislation and the institutional requirements.

## Author Contributions

YF-H, AM, and LS conceived the study, designed the study, and authored the manuscript. YL and MQ performed data extraction. YF-H and WL conducted the analysis. AM, MC, BG, JMK, MD, EB, and JK contributed to data interpretation, discussion, and manuscript preparation. All authors contributed to the article and approved the submitted version.

## Funding

This work was supported by the National Institutes of Health: the National Institute on Aging (K08 AG054727, UH3 AG060626, R01 AG072911, R21 AG061307, R21 NR018500).

## Conflict of Interest

Consulting fees, payments, honoraria, or support for attending meetings: MC: Board fee from Haven Behavioral Health Care Board; SUNY Downstate School of Medicine Department of Medicine Grand Rounds; Maimonides Medical Center Department of Medicine Grand Rounds; Board of Governors of American College of Physicians 2018–2021 meetings; JK: Consultant to or receives honoraria: Alkermes, Allergan, Dainippon Sumitomo, H. Lundbeck, Indivior, Intracellular Therapies, Janssen Pharmaceutical, Johnson & Johnson, LB Pharmaceuticals, Merck, Minerva, Neurocrine, Novartis Pharmaceuticals, Otsuka, Reviva, Roche, Saladex, Sunovion, Takeda, Teva Grant Support: Otsuka, Lundbeck, Sunovion, Vanguard Research Group LB Pharmaceuticals, and North Shore Therapeutics; Participation on Data Safety Monitoring Board or Advisory Board: JK: Teva and Novartis; EB: PCORI Eugene Washington Award advisory panel member. Leadership or fiduciary role: MD: Society of Behavioral Medicine, President and past President (unpaid). EB: Society of Behavioral Medicine: nominating committee; annual program committee (unpaid). Associate editor, Translational Behavioral Medicine (unpaid). Stock or stock options: YF-H co-owns stock and stock options from Regeneron Pharmaceuticals. The remaining authors declare that the research was conducted in the absence of any commercial or financial relationships that could be construed as a potential conflict of interest.

## Publisher's Note

All claims expressed in this article are solely those of the authors and do not necessarily represent those of their affiliated organizations, or those of the publisher, the editors and the reviewers. Any product that may be evaluated in this article, or claim that may be made by its manufacturer, is not guaranteed or endorsed by the publisher.
