# Supplementary material for: Psychotropic Medication Use Is Associated With Greater 1-Year Incidence of Dementia After COVID-19 Hospitalization
Source: Front Med (Lausanne). 2022 Mar 18;9:841326. doi: 10.3389/fmed.2022.841326 (PMC8972194; doi:10.3389/fmed.2022.841326)
Supplement: Supplementary file 1 [file Data_Sheet_1.docx]

**Supplementary content**

**Psychotropic medication use is associated with greater one-year incidence of dementia after COVID-19 hospitalization**

Yun Freudenberg-Hua, MD^1,2,3^, Alexander Makhnevich, MD ^2,3,4^, Wentian Li, PhD^2^, Yan Liu, PhD^2^, Michael Qiu, MD^2^, Allison Marziliano, PhD^2,3,4^, Maria Carney, MD^2,3,4^, Blaine Greenwald, MD^1,2,3^, John M. Kane, MD^1,2,3^, Michael Diefenbach, PhD^2,3,4^, Edith Burns, MD^2,3,4^, Jeremy Koppel, MD^1,2,3^, and Liron Sinvani, MD^2,3,4^

Corresponding Author: Yun Freudenberg-Hua

Affiliations:

1. Department of Psychiatry, Zucker Hillside Hospital, Northwell Health, Glen Oaks, NY, USA
2. The Feinstein Institutes for Medical Research, Manhasset, NY, USA
3. Donald and Barbara Zucker School of Medicine at Hofstra/Northwell, 500 Hofstra Boulevard, Hempstead, NY, USA
4. Department of Medicine, Donald and Barbara Zucker School of Medicine at Hofstra Northwell

**eMethods**

**eTable 1. Strengthening the Reporting of Observational Studies in Epidemiology (STROBE) checklist**

**eTable 2. Psychotropic medication list**

**eTable 3. Multivariable Cox Regression for Incident Dementia in Older Adults Hospitalized With COVID-19 (n=1755)**

**eTable 4. Sensitivity analysis in 423 patients with neurological or psychiatric diagnoses**

**eTable 5. Patient Demographics and Characteristics Stratified by Availability of Follow-up Data within the Health System (unadjusted)**

**eFigure 1. Power analysis for required sample size for association analysis with individual psychotropic medications**

**eFigure 2. Random Forest Variable Importance for Predicting post-COVID Dementia**

**eFigure 3. LASSO Regression Coefficient Profile**

**eMethods**

To perform additional validation for the results of multiple logistic regression, we applied Least Absolute Shrinkage and Selection Operator (LASSO) regression to minimize overfitting of variables as well as potential collinearity of variables (1). In addition, the sequence of variable selection was informative as it indicated the importance of variable contribution as well as fitting of the model (2). Different from a regular multiple regression where the coefficients are fitted to minimize the square-difference between the output values and regression fitting model, LASSO minimizes the square-difference, which is subject to the condition that the sum of all absolute value of variable coefficients is smaller than a given threshold. When the threshold is small enough, all variable coefficients are forced to be zero, and when the threshold gradually increases, variables are selected one after another. The first variable selected is assumed to be more important than those subsequently selected.

**eTable 1. Strengthening the Reporting of Observational Studies in Epidemiology (STROBE) checklist**

|  | | Item No | Recommendation | Page No |
| --- | --- | --- | --- | --- |
| **Title and abstract** | | 1 | (*a*) Indicate the study’s design with a commonly used term in the title or the abstract | 1,2 |
|  |  |  | (*b*) Provide in the abstract an informative and balanced summary of what was done and what was found | 2 |
| Introduction | | | | |
| Background/rationale | | 2 | Explain the scientific background and rationale for the investigation being reported | 4 |
| Objectives | | 3 | State specific objectives, including any prespecified hypotheses | 5 |
| Methods | | | | |
| Study design | | 4 | Present key elements of study design early in the paper | 5,6,7,8 |
| Setting | | 5 | Describe the setting, locations, and relevant dates, including periods of recruitment, exposure, follow-up, and data collection | 5,6 |
| Participants | | 6 | (*a*) Give the eligibility criteria, and the sources and methods of selection of participants. Describe methods of follow-up | 5,6 |
|  |  |  | (*b*) For matched studies, give matching criteria and number of exposed and unexposed | NA |
| Variables | | 7 | Clearly define all outcomes, exposures, predictors, potential confounders, and effect modifiers. Give diagnostic criteria, if applicable | 6,7 |
| Data sources/ measurement | | 8* | For each variable of interest, give sources of data and details of methods of assessment (measurement). Describe comparability of assessment methods if there is more than one group | 6,7 |
| Bias | | 9 | Describe any efforts to address potential sources of bias | 7,8 |
| Study size | | 10 | Explain how the study size was arrived at | 5,6,9 Figure 1 |
| Quantitative variables | | 11 | Explain how quantitative variables were handled in the analyses. If applicable, describe which groupings were chosen and why | 8 |
| Statistical methods | | 12 | (*a*) Describe all statistical methods, including those used to control for confounding | 8 |
|  |  |  | (*b*) Describe any methods used to examine subgroups and interactions | 8 |
|  |  |  | (*c*) Explain how missing data were addressed | 8 |
|  |  |  | (*d*) If applicable, explain how loss to follow-up was addressed | 12 |
|  |  |  | (*e*) Describe any sensitivity analyses | 8 |
| Results | | | |  |
| Participants | | 13* | (a) Report numbers of individuals at each stage of study—eg numbers potentially eligible, examined for eligibility, confirmed eligible, included in the study, completing follow-up, and analysed | 9,12 |
|  |  |  | (b) Give reasons for non-participation at each stage | 9,12; Figure 1 |
|  |  |  | (c) Consider use of a flow diagram | Figure 1 |
| Descriptive data | | 14* | (a) Give characteristics of study participants (eg demographic, clinical, social) and information on exposures and potential confounders | Table 1 |
|  |  |  | (b) Indicate number of participants with missing data for each variable of interest | Table 1 |
|  |  |  | (c) Summarise follow-up time (eg, average and total amount) | 5,9 |
| Outcome data | | 15* | Report numbers of outcome events or summary measures over time | NA |
| Main results | 16 | (*a*) Give unadjusted estimates and, if applicable, confounder-adjusted estimates and their precision (eg, 95% confidence interval). Make clear which confounders were adjusted for and why they were included | | 9,10,11; Table 1; Table 2 |
|  |  | (*b*) Report category boundaries when continuous variables were categorized | | NA |
|  |  | (*c*) If relevant, consider translating estimates of relative risk into absolute risk for a meaningful time period | | NA |
| Other analyses | 17 | Report other analyses done—eg analyses of subgroups and interactions, and sensitivity analyses | | 11,12,13; Table 3; eTables 3,4,5 |
| Discussion | | | | |
| Key results | 18 | Summarise key results with reference to study objectives | | 13,14 |
| Limitations | 19 | Discuss limitations of the study, taking into account sources of potential bias or imprecision. Discuss both direction and magnitude of any potential bias | | 18 |
| Interpretation | 20 | Give a cautious overall interpretation of results considering objectives, limitations, multiplicity of analyses, results from similar studies, and other relevant evidence | | 13,19 |
| Generalisability | 21 | Discuss the generalisability (external validity) of the study results | | 19 |
| Other information | | | | |
| Funding | 22 | Give the source of funding and the role of the funders for the present study and, if applicable, for the original study on which the present article is based | | 20 |

**eTable 2. Psychotropic medication list**

| **Antipsychotic** | **Antidepressant** | **Mood Stabilizer/ Anticonvulsant** | **Benzodiazepine** | **Antiparkinson** | **Dementia Medication** |
| --- | --- | --- | --- | --- | --- |
| aripiprazole | citalopram | valproic acid | alprazolam | amantadine | memantine |
| asenapine | escitalopram | valproate | chlordiazepoxide | entacapone | donepezil |
| brexpiprazole | fluoxetine | lamotrigine | clonazepam | opicapone | galantamine |
| cariprazine | fluvoxamine | carbamazepine | clorazepate | pramipexole | rivastigmine |
| chlorpromazine | paroxetine | phenytoin | diazepam | ropinirole |  |
| clozapine | sertraline | ethosuximide | estazolam | rotigotine |  |
| droperidol | bupropion | levetiracetam | flurazepam | bromocriptine |  |
| fluphenazine | mirtazapine | topiramate | lorazepam | levodopa |  |
| haloperidol | nefazodone | phenobarbital | midazolam | carbidopa |  |
| iloperidone | trazodone | vigabatrin | triazolam | cabergoline |  |
| loxapine | vilazodone | lithium | quazepam | safinamide |  |
| lumateperone | vortioxetine | oxcarbazepine | oxazepam | profenamine |  |
| lurasidone | phenelzine |  | temazepam | rasagiline |  |
| molindone | selegiline |  | hydroxyzine |  |  |
| olanzapine | tranylcypromine |  |  |  |  |
| paliperidone | desvenlafaxine |  |  |  |  |
| perphenazine | duloxetine |  |  |  |  |
| pimavanserin | venlafaxine |  |  |  |  |
| pimozide | milnacipran |  |  |  |  |
| prochlorperazine | levomilnacipran |  |  |  |  |
| quetiapine | amitriptyline |  |  |  |  |
| risperidone | desipramine |  |  |  |  |
| thioridazine | doxepine |  |  |  |  |
| thiothixene | imipramine |  |  |  |  |
| trifluoperazine | nortriptyline |  |  |  |  |
| ziprasidone | amoxapine |  |  |  |  |
|  | clomipramine |  |  |  |  |
|  | maprotiline |  |  |  |  |
|  | trimipramine |  |  |  |  |
|  | protriptyline |  |  |  |  |

| **eTable 3. Multivariable Cox Regression for Incident Dementia in Older Adults Hospitalized With COVID-19 (n=1755)** | | | | | | |
| --- | --- | --- | --- | --- | --- | --- |
|  | **Model 1** | | | **Model 2** | | |
| *Predictors* | *HR* | *95% CI* | *p* | *HR* | *95% CI* | *p* |
| Age | 1.02 | 1.00  – 1.05 | 0.09 | 1.03 | 1.01 – 1.06 | **0.01** |
| Sex (ref="Female") | 0.91 | 0.64 – 1.30 | 0.60 | 0.9 | 0.93 – 1.29 | 0.57 |
| Race, Asian (ref ="White") | 1.36 | 0.66 – 2.80 | 0.40 | 1.36 | 0.66 – 2.81 | 0.40 |
| Race, Black (ref ="White") | 0.81 | 0.50 – 1.30 | 0.38 | 0.76 | 0.47 – 1.22 | 0.25 |
| Race, Other/unknown, (ref ="White") | 1.28 | 0.71 – 2.31 | 0.40 | 1.34 | 0.74 – 2.45 | 0.34 |
| Ethnicity (ref="Hispanic") | 1.15 | 0.60 – 2.21 | 0.68 | 1.14 | 0.59 – 2.22 | 0.69 |
| Smoking | 0.93 | 0.48 – 1.80 | 0.82 | 0.96 | 0.50 – 1.87 | 0.91 |
| BMI | 0.95 | 0.92 – 0.98 | **0.002** | 0.95 | 0.91 – 0.98 | **<.001** |
| Comorbidity Index | 1.05 | 0.99 – 1.12 | 0.10 | 1.06 | 1.00 – 1.13 | **0.05** |
| Delirium | 3.03 | 2.07 – 4.44 | **<.001** | 2.61 | 1.75 – 3.90 | **<.001** |
| MEWS | 1.09 | 0.92 – 1.28 | 0.32 | 1.09 | 0.93 – 1.28 | 0.29 |
| Highest Oxygen Delivery | 1.14 | 0.88 – 1.49 | 0.32 | 1.17 | 0.90 – 1.53 | 0.24 |
| Any Psychotropic | 2.48 | 1.73 – 3.58 | **<.001** | ___ | ___ | ___ |
| Antipsychotic | ___ | ___ | ___ | 2.28 | 1.41 – 3.97 | **0.003** |
| Mood Stabilizer/ Anticonvulsant | ___ | ___ | ___ | 2.32 | 1.30 – 4.16 | **0.005** |
| Antidepressant | ___ | ___ | ___ | 1.23 | 0.80 – 1.88 | 0.34 |
| Benzodiazepine | ___ | ___ | ___ | 1.22 | 0.66 – 2.24 | 0.53 |
| Antiparkinson | ___ | ___ | ___ | 1.36 | 0.58 – 3.17 | 0.48 |

***Note*.** Time to event is defined as days between index COVID-19 admission and date of dementia diagnosis for patients with incident dementia and as days between index COVID-19 admission and date of last follow-up for patients without incident dementia. HR: Hazard ratio; For binary variables Smoking, Delirium, Any Psychotropic, Antipsychotic, Mood Stabilizer/Anticonvulsant, Antidepressant, Benzodiazepine, and Antiparkinson, the reference values were “No” or “not exposed”. Ethnicity: Hispanic or not-Hispanic; Smoking: never smoker or current/past smoker; BMI: body mass index; Comorbidity Index: Charlson Comorbity Index; MEWS: Modified Early Warning Score. Highest Oxygen Delivery: Highest level of oxygen support during index hospitalization; Any Psychotropic: medications including all subcategories of psychotropic medications: antipsychotics, antidepressants, benzodiazepines, mood stabilizers/anticonvulsants (including lithium), and Parkinson’s disease medications (Antiparkinson).

**eTable 4. Sensitivity analysis in 423 patients with neurological or psychiatric diagnoses**

|  | **Incident Dementia** | | |
| --- | --- | --- | --- |
| *Predictors* | *Odds Ratios* | *95% CI* | *p* |
| Age | 1.02 | 0.96 – 1.07 | 0.56 |
| Sex (ref="Female") | 1.09 | 0.55 – 2.20 | 0.80 |
| Race, Asian (ref ="White") | 4.05 | 0.91 – 15.97 | **0.05** |
| Race, Black (ref ="White") | 1.18 | 0.48 – 2.76 | 0.71 |
| Race, Other/unknown, (ref ="White") | 0.69 | 0.18 – 2.25 | 0.56 |
| Ethnicity (ref="Hispanic") | 2.29 | 0.54 – 9.49 | 0.25 |
| Smoking | 0.11 | 0.01 – 0.65 | **0.05** |
| BMI | 0.93 | 0.87 – 0.99 | **0.02** |
| Comorbidity Index | 1.04 | 0.93 – 1.15 | 0.49 |
| Delirium | 2.80 | 1.34 – 5.82 | **0.006** |
| MEWS | 0.91 | 0.66 – 1.23 | 0.53 |
| Highest Oxygen Delivery | 1.61 | 0.98 – 2.63 | 0.06 |
| Any Psychotropic | 3.09 | 1.52 – 6.57 | **0.002** |

***Note.*** For binary variables Smoking, Delirium, Any Psychotropic, Antipsychotic, Mood Stabilizer/Anticonvulsant, Antidepressant, Benzodiazepine, and Antiparkinson, the reference values were “No”. Ethnicity: Hispanic or not-Hispanic; Smoking: never smoker or current/past smoker; BMI: body mass index; Comorbidity Index: Charlson Comorbity Index; MEWS: Modified Early Warning Score. Highest Oxygen Delivery: Highest level of oxygen support during index hospitalization; Delirium: Delirium during index admission; Any Psychotropic: pre-COVID exposure to psychotropic medications.

| **eTable 5. Patient Demographics and Characteristics Stratified by Availability of Follow-up Data within the Health System (unadjusted)** | | | |
| --- | --- | --- | --- |
|  | Follow-up within Health System | |  |
|  | No | Yes | P |
| N | 1219 | 1995 |  |
| Gender (% Male) | 636 (52.2) | 1091 (54.7) | 0.177 |
| Age (mean (SD)) | 76.42 (8.55) | 76.09 (7.97) | 0.257 |
| Race (%) |  |  | <0.001 |
| Asian | 107 (8.8) | 126 (6.3) |  |
| Black | 255 (20.9) | 472 (23.7) |  |
| Other_or_unknown | 351 (28.8) | 454 (22.8) |  |
| White | 506 (41.5) | 943 (47.3) |  |
| Ethnicity (% Non-Hispanic) | 910 (80.8) | 1615 (84.8) | 0.006 |
| Comorbidity.Index (mean (SD)) | 2.61 (2.41) | 3.46 (2.86) | <0.001 |
| BMI (mean (SD)) | 26.81 (5.80) | 27.45 (5.93) | 0.005 |
| Smoking (%) | 51 (4.4) | 120 (6.2) | 0.038 |
| MEWS (mean (SD)) | 3.60 (1.21) | 3.55 (1.18) | 0.247 |
| Highest Level of Oxygen (mean (SD)) | 1.50 (1.58) | 1.60 (1.75) | 0.11 |
| Delirium (%) | 208 (17.1) | 333 (16.7) | 0.822 |
| Pre-COVID Dementia (%) | 177 (14.5) | 240 (12.0) | 0.047 |
| Any Psychotropic (%) | 310 (25.4) | 616 (30.9) | 0.001 |
| Antipsychotic (%) | 102 (8.4) | 175 (8.8) | 0.74 |
| Antidepressant (%) | 147 (12.1) | 324 (16.2) | 0.001 |
| Benzodiazepine (%) | 54 (4.4) | 119 (6.0) | 0.073 |
| Mood Stabilizer/ Anticonvulsant (%) | 58 (4.8) | 120 (6.0) | 0.152 |
| Antiparkinson (%) | 34 (2.8) | 61 (3.1) | 0.742 |

***Note.*** Ethnicity: Hispanic or not-Hispanic; Smoking is defined as current or past smoker; BMI: body mass index; Comorbidity Index: Charlson Comorbidity Index without the age component; MEWS: Modified Early Warning Score. Highest Level of Oxygen: the highest level of oxygen support during index hospitalization. “0”: room air or nasal cannula; “1”: Venturi Mask, nonrebreather, high flow, noninvasive positive-pressure ventilation; “2”: invasive mechanical ventilation. Pre-COVID Dementia: Having dementia diagnosis or cognitive impairment or being prescribed a medication used for dementia at the time of index COVID-19 admission. Any Psychotropic: medications including all subcategories of psychotropic medications: antipsychotics, antidepressants, benzodiazepines, mood stabilizers/anticonvulsants (including lithium), and Parkinson’s disease medications (Antiparkinson).

**eFigure 1. Power analysis for required sample size for association analysis with individual psychotropic medications**

***Note.*** Power analysis for testing association of an individual medication is performed using two-sample test for proportions with unequal sample sizes: pwr.2p2n.test(h =ES.h(p1, p2), n1, power, sig.level). Abbreviations: “p1” is the dementia incidence rate in patients without exposure to pre-COVID psychotropic medication. “p2” is the dementia incidence rate in patients with pre-COVID exposure to a specific psychotropic medication. “n1” is the number of patients not exposed to pre-COVID psychotropic medication. “n2” is the number of patients exposed to a specific psychotropic medication.

**eFigure 2. Random Forest Variable Importance for Predicting post-COVID Dementia**

**
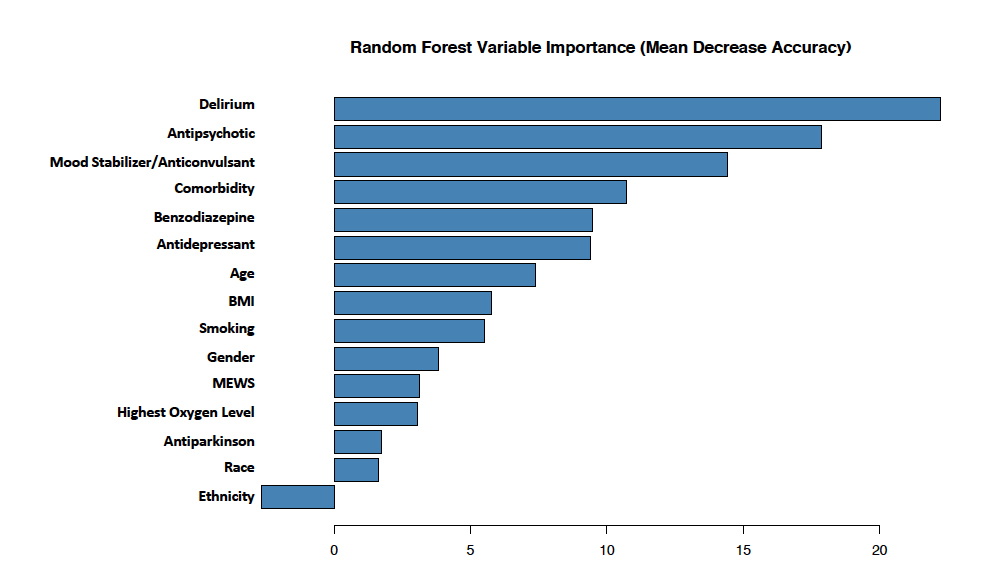
**

***Note*.** The x-axis represents the mean decrease in classification accuracy for a variable, which is the normalized difference of the classification accuracy when that variable is included as observed versus when the values of that variable have been randomly permuted. The variables are presented on the y-axis in decreasing order of importance for predicting post-COVID dementia. Abbreviations: BMI: body mass index; MEWS: Modified Early Warning Score.

**eFigure 3. LASSO Regression Coefficient Profile**

**
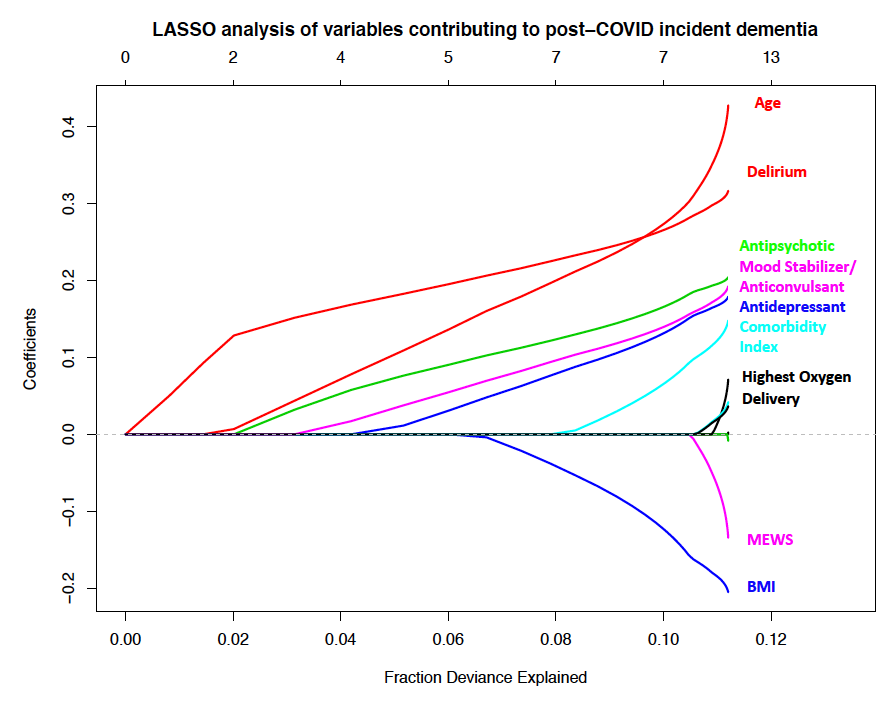
**

***Note.*** LASSO coefficient profile for the predictor variables included in **Model 2** of main results. To ensure all variables are comparable, in particular between binary input variables and continuous variables such as age, we standardize each variable to have zero mean and unity standard deviation.

The x-axis of a LASSO coefficient profile is a variable that indicates the relaxation of constraint on regression coefficient, from strong constrain at left to weaker constrain to the right. Here we use the percentage reduction of deviance (-2*log(likelihood)) from that of the null model (without using any predictor variable). The y-axis is the regression coefficient for a variable. Abbreviations: BMI: body mass index; MEWS: Modified Early Warning Score. Highest Oxygen Delivery: Highest level of oxygen support during index hospitalization.

**References**

1.Taylor J, Tibshirani RJ. Statistical learning and selective inference. Proc Natl Acad Sci U S A. 2015;112(25):7629-34.

2.Pavlou M, Ambler G, Seaman S, De Iorio M, Omar RZ. Review and evaluation of penalised regression methods for risk prediction in low-dimensional data with few events. Stat Med. 2016;35(7):1159-77.
